# Double traumatic diaphragmatic injury: A case report

**DOI:** 10.1016/j.ijscr.2019.07.030

**Published:** 2019-07-19

**Authors:** Dario Iadicola, Massimo Branca, Massimo Lupo, Eugenia Maria Grutta, Stefano Mandalà, Gianfranco Cocorullo, Antonino Mirabella

**Affiliations:** a Department of Surgical, Oncological and Stomatologic Disciplines (Di.Chir.On.S.), University of Palermo, Palermo, Italy; b Department of General and Emergency Surgery - Azienda Ospedali Riuniti Villa Sofia-Cervello, Palermo, Italy; c University of Palermo, Palermo, Italy; d Casa di Cura Noto-Pasqualino, Palermo, Italy

**Keywords:** Tramautic diaphragmatic hernia, Diaphragmatic injury, Titanized mesh, Trauma, Abdomen, Thorax, Laparoscopy

## Abstract

**Introduction:**

Traumatic diaphragmatic injuries are rare complications resulting from a thoracic-abdominal blunt or penetrating trauma. Left-sided diaphragmatic injuries are more commonly reported in literature. Bilateral injuries are extremely rare, occurring in about 3% of the patients and just few cases reported in literature. Traumatic diaphragmatic hernias are definitely a marker of a severe trauma, in fact diaphragmatic injuries are often related to thoracic and abdominal organs injuries. Sometimes the classic clinical signs and symptoms of diaphragmatic injuries may initially not be present so that definitive evaluation is delayed or even missed.

**Case report:**

A 62-years old woman was admitted in Emergency Department after a pedestrian accident. A whole-body CT scan showed multiple fractures (ribs, pelvic and vertebral) but no organ injury. The next CT detected a left-sided posterior diaphragmatic hernia involving transverse colon. Thus we performed an explorative laparoscopy and found a double diaphragmatic injury. A primary repair with non-absorbable sutures and a prosthetic titanized patch was performed.

## Highlights

• Diaphragmatic injuries are rare complications from trauma.
• Bilateral diaphragmatic injuries are extremely rare and just a few cases are reported.
• Sometimes the diagnosis is delayed or even missed.
• Both primary repair or mesh repair are safe and feasible.
• The use of a polypropylene mesh with titanized surface has not been attempted before.

## 1 Introduction

Traumatic diaphragmatic injuries are rare complications resulting from a thoracic-abdominal blunt or penetrating trauma. Since diaphragmatic injuries are quite uncommon in trauma, with a reported incidence in literature between 0.8% and 8% [1], the true incidence of abdominal organ herniation is unknown, also because many cases remain undiagnosed. The blunt vs. penetrating trauma ratio is widely variable geographically [[1], [2], [3]]. Male people, especially aging from 30 to 45 y.o. are most frequently involved [1,3,5]. According to AAST (American Association for the Surgery of Trauma) Classification (Table 1), diaphragmatic injuries are clustered into 5 grades: I (Contusion); II (Laceration <2 cm); III (Laceration 2–10 cm); IV (Laceration >10 cm with tissue loss <25 cm^2^); V (Laceration with tissue loss >25 cm^2^) [4]. Left-sided diaphragmatic injuries are more commonly reported in literature (60–70%) [3,5], probably due to the protective effect of the liver on the right side or a left-side embryologic weakness [1,3,5], and mostly get complicated involving stomach, colon and spleen [5]. On the other hand, right-sided wounds, accounting about 30–40% [3,5], requires higher energy (i.e. high speed collisions) and involve liver or colon. Bilateral injuries are extremely rare, occurring in about 3% of the patients and just a few cases reported in literature [1]. Blunt trauma typically produces large radial tears (5–15 cm), most often located in the left postero-lateral region of the diaphragm [5]. On the other hand, penetrating trauma usually generates small linear incisions or holes (<2 cm in size) [5]. An abrupt change in intra-abdominal pressure (i.e. motor vehicle crashes; falls from height), shearing and avulsion are the main mechanisms of diaphragmatic injuries in blunt trauma [1,5]. Furthermore the higher pressure gradient across diaphragm contributes to the initial injury and to the herniation of abdominal organs as well. Hence, because of their own pathophysiology, traumatic diaphragmatic hernias can be considered definitely a marker of a severe trauma. In fact diaphragmatic injuries are often related to thoracic and abdominal organs injuries (aorta, kidney, hollow viscera, liver, lung, spleen, pelvic and rib fractures) and severe complications (DVT/PE, hemo-pneumothorax, pneumonia, respiratory distress, sepsis), with a high mortality rate reported in literature (20%) [1]. Nevertheless sometimes the classic clinical signs and symptoms of diaphragmatic injuries may initially not be present or associated damages may be so severe that definitive evaluation is delayed or even missed [1,3,5]. Thus the diaphragmatic wound will become larger and herniation of abdominal organs more likely, producing respiratory distress or bowel obstruction or strangulation [3,5]. Radiological imaging plays a central role in diagnosis. Plain chest X-ray and FAST ultrasound, although represent the most accessible and first line imaging modality in the trauma patients, have a poor accuracy (nonspecific alterations in only 20%–50% of the patients - i.e. interruption of diaphragm silhouette, hemidiaphragm elevation, costo-phrenic sulcus obliteration or distorted diaphragmatic profile) [5,6]. The only direct sign is represented by the visualization of herniated bowels or the nasogastric tube into the thoracic cavity [5,6]. For this reason, whole-body contrast CT scan with multiplanar reconstructions is nowadays the imaging modality of choice. Thanks to its high accuracy (60%–90% sensitivity and 70%–100% specificity) [5,6], it is able to directly detect diaphragmatic lesions, herniated bowels or indirect signs (i.e. “dangling diaphragm sign”, “collar sign” and “hump and band” sign) [6]. When equivocal findings on imaging studies are present and conservative management (NOM) is not suitable, exploratory laparoscopy achieve 90–100% sensibility and specificity in detecting diaphragmatic injuries [1,5,7]. Furthermore in selected patients, laparoscopic repair of diaphragmatic injuries is safe and feasible when performed by highly skilled surgeons [5,[7], [8], [9], [10]].

**Table 1:** Diaphragm Injury Scale (from Moore EE et al. Organ injury scaling. IV: Thoracic vascular, lung, cardiac, and diaphragm. J Trauma. 1994 Mar;36(3):299–300).

Diaphragm Injury Scale	
Grade*	Description of injury
I	Contusion
II	Laceration <2 cm
III	Laceration 2–10 cm
IV	Laceration >10 cm with tissue loss (<25 cm^2^)
V	Laceration with tissue loss >25 cm^2^

* Advance one grade for bilateral injuries up to grade III.

## 2 Case report

**Fig. 1:**
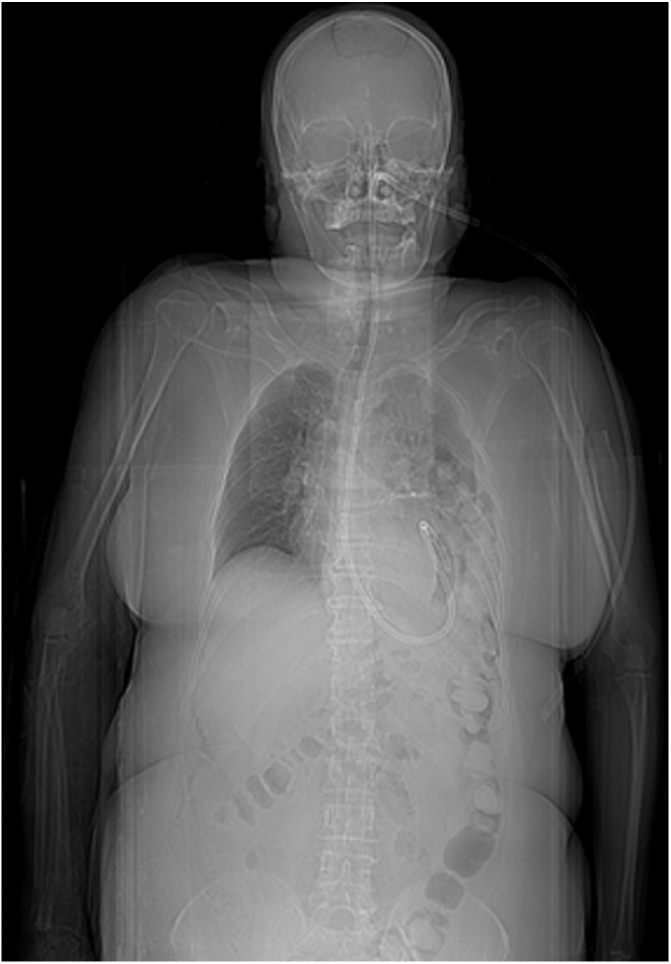
Whole-body non-contrast CT scan (scout view): detection of left-sided posterior diaphragmatic hernia involving transverse colon.

**Fig. 2:**
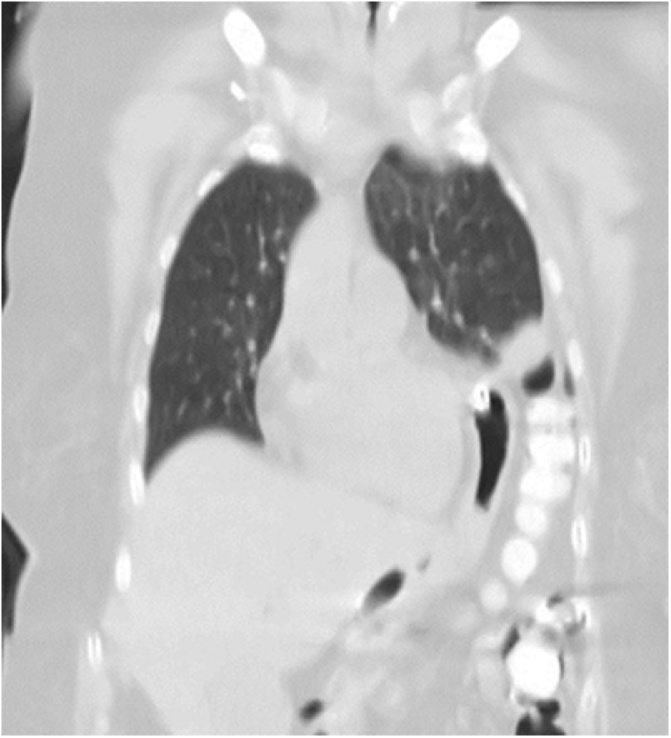
Whole-body oral-contrast CT scan (coronal view with pulmonary window).

A 62-years old woman was admitted to Emergency Department in our hospital after a pedestrian accident (blunt head-thoracic-abdominal trauma). After resuscitation and supportive therapy, she underwent a whole-body intravenous contrast CT scan and admitted in Trauma Center Department (Level I) in the same hospital. The CT scan reported a frontal and basilar subarachnoid hemorrhage, right-sided fronto-temporo-parietal subdural effusion, multiple bilateral rib fractures (anterior arch of III-IV-V-VI-VII-VIII ribs on the right side and anterior arch of III-IV-V ribs on the left side), multiple pelvic fractures (both acetabula, sacrum and pubic rami bilaterally) and multiple lumbar vertebral fractures (L2-L3-L4 transverse process) - estimated ISS (Injury Severity Scale) 34 (Table 2, Table 3). No pneumothorax, pneumoperitoneum or thoracic-abdominal effusion or organ injury were detected. Thus orotracheal intubation was performed and a supportive therapy was established. In the days after, the clinical conditions improved and she was weaned from ventilator and the supportive therapy was reduced. Other whole-body non-contrast CT scans were performed with no change excluding a moderate bilateral pleural effusion. The next CT scan detected instead a left-sided posterior diaphragmatic hernia involving transverse colon (Fig. 1), although no sign of bowel obstruction or strangulation appeared. Hence, a further thorax-abdomen oral-contrast CT scan was request (Fig. 2) and confirmed our previous suspect. Nevertheless the patient was in pretty good condition, not in respiratory distress or bowel obstruction.

**Table 2:** Abbreviated Injury Scale - AIS (from Copes WS et al. Progress in Characterising Anatomic Injury, In Proceedings of the 33rd Annual Meeting of the Association for the Advancement of Automotive Medicine, Baltimore, MA, USA 205–218).

Abbreviated Injury Scale (AIS)	
AIS Score	Grade
1	Minor
2	Moderate
3	Serious
5	Critical
6	Unsurvivable

**Table 3:** ISS Score: A² + B² + C² (from Baker SP et al. The injury severity score: a method for describing patients with multiple injuries and evaluating emergency care. J Trauma. 1974 Mar;14(3):187–96).

Injury Severity Scale (ISS)	
Region	AIS Score
Head and neck	1-2-3-4-5-6
Face	1-2-3-4-5-6
Chest	1-2-3-4-5-6
Abdomen	1-2-3-4-5-6
Extremity (including pelvis)	1-2-3-4-5-6
External	1-2-3-4-5-6

## 3 Surgical technique

**Fig. 3:**
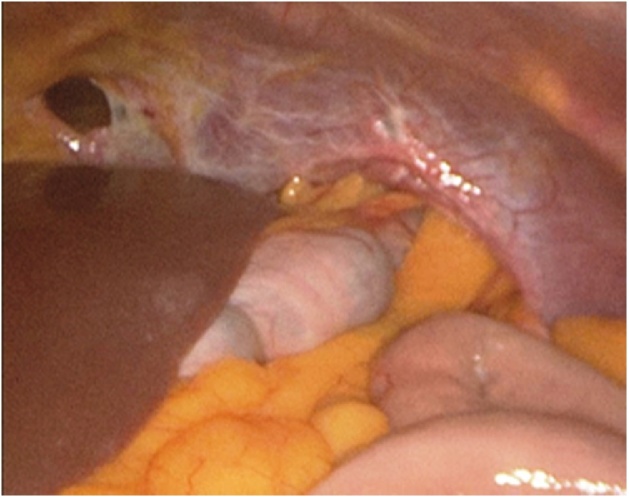
Double diaphragmatic injury.

**Fig. 4:**
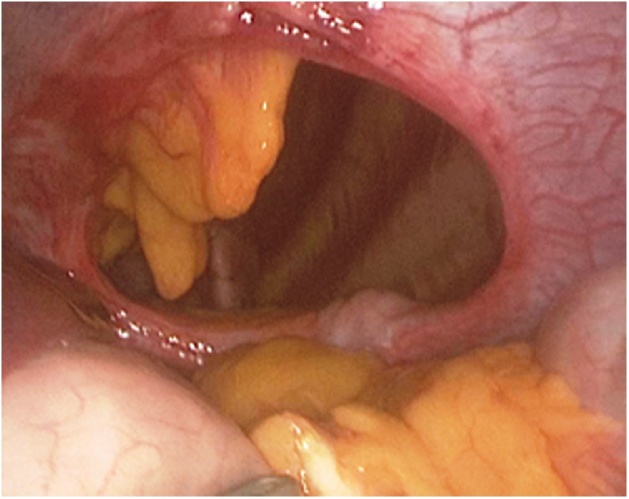
Left-sided postero-lateral hernia.

**Fig. 5:**
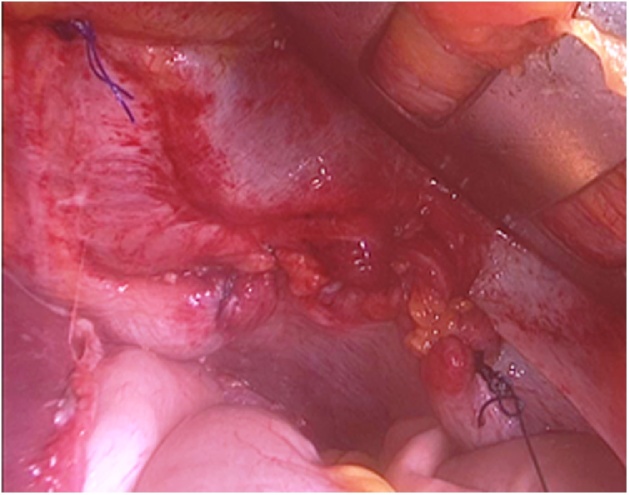
Defects repaired with non-absorbable polypropylene suture.

**Fig. 6:**
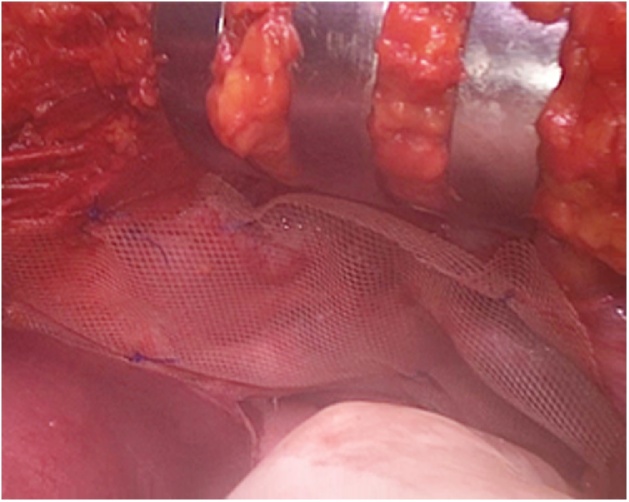
Polypropylene mesh with titanized surface (TiMesh strong^®^, PFM Medical AG, Cologne, Germany) attached to the diaphragm.

We decided to perform an exploratory laparoscopy using a 10 mm peri-umbilical port for camera and two lateral ports (5 mm port in right flank and 10 mm port in right flank). At first we performed a laparoscopic assessment of intra-abdominal organs, after that we noted a double diaphragmatic injury (Fig. 3) (retrocostal-xiphoid and left-sided postero-lateral). The retrocostal-xiphoid injury was 3 cm wide - AAST grade III - and had no content. On the other hand, the left-sided postero-lateral injury was bigger (width: 8 cm - AAST grade III) with the edges looking fibrotic (Fig. 4) and the greater omentum, the stomach and the transverse colon were displaced inside. Thus we reduced the hernia by gentle traction back to peritoneal cavity, exposing the left lung on the background. Furthermore we performed a little left exploratory thoracoscopy through the bigger defect and found no signs of lung or heart injury or hemorrhage. We inserted also a 32 Fr chest drainage in the left hemithorax, in order to evacuate the pneumothorax [11]. We continued the abdominal exploration and no other organ (i.e. liver or spleen) were injuried. Since the singularity, complexity and the plurality of those diaphragmatic injuries and the proximity to pericardium, we decided to convert to open surgery through an upper midline laparotomy. Thus we repaired the defects with non-absorbable polypropylene suture (Prolene^®^ 2-0, Ethicon Inc., Somerville, USA) in a running fashion (Fig. 5). Furthermore we decided to use a prosthetic mesh to cover the defects and strengthen the diaphragm weakness. We used a 15 × 10 cm lightweight polypropylene mesh with covalently bonded titanized surface (TiMesh strong^®^, PFM Medical AG, Cologne, Germany) attached to the diaphragm with non-absorbable polypropylene suture (Prolene^®^ 3-0, Ethicon Inc., Somerville, USA) in an interrupted fashion (Fig. 6). In the end we achieved emostasis and inserted a 28 Fr drainage in the splenic lodge. Later the patient was admitted to ICU for close supervision, intensive treatment and support. The post-operative course was regular and in post-operative day 4, she was admitted to our Department. Here, after many multi-specialist revaluations and lab values normalization, the patient was discharged in post-operative day 17, for rehabilitation.

## 4 Discussion

Bilateral or multiple diaphragmatic injuries are exceptionally rare conditions, accounting about 3% all diaphragmatic wounds [1]. Even though laparoscopic repair of diaphragmatic injuries is safe and feasible when performed by highly skilled surgeons, no attempt to repair complex injuries laparoscopically should be made, because of the potential risk of hazardous complications [7]. Since the retrocostal-xiphoid defect was opened into the mediastinum and the beating heart was visible through the defect, we decided for conversion to open surgery in order to avoid a pericardium lesion and to achieve a safe and adequate repair of the diaphragm wounds [12]. Since the edges of the bigger wound looked fibrotic like in a chronic defect, although not detected to a previous CT scan, we decided to cover the defects and further strengthen the diaphragm weakness. In fact mesh repair is usually recommended when the defect is too large for primary repair (AAST grade IV-V) or in case of a chronic defect. In this case a primary tension-free repair cannot be achieved and a suture line dehiscence is more likely [5]. Even if the use of nonabsorbable prosthetic materials (e.g. polytetrafluoro-ethylene, polyethylene) is fully established, biologic meshes are an acceptable alternative, inducing limited inflammatory response and minimizing adhesion formation [13,14]. We selected this lightweight polypropylene mesh with titanized surface due to its established lower risk of inflammation, better tolerability, capability of less scar formation and less shrinking of the mesh [15,16]. No record of previous use of this type of mesh for diaphragmatic injuries has been found in literature. This patient is currently under a strict follow-up program for 6 months, up to now she has not experienced any complication or complained any problem. A recent CT scan detected no evidence of recurrence, eventration or obstruction. Further studies and a longer follow-up program are required to establish the successful and feasible use of titanized meshes.

## 5 Disclaimer

The paper has been reported in line with the SCARE criteria [17]. This research has received no external funding. The authors declare no conflict of interest.

## Funding

The authors declare no external funding and no sponsor involvement.

## Ethical approval

Ethical approval exemption was given for this study.

## Consent

Written consent was obtained by the patient for publishing this case report.

## Author contribution

Dario Iadicola M.D. designed this study and wrote this article. Antonino Mirabella M.D. contributed in designing this study. Massimo Branca M.D. PH.D., Massimo Lupo M.D., Eugenia Maria Grutta M.D., Stefano Mandalà, M.D. and Gianfranco Cocorullo M.D. PH.D. contributed in data collection and analysis.

## Registration of research studies

This study has not been registered.

## Guarantor

Dario Iadicola M.D. and Antonino Mirabella M.D. accept full responsibility for this work and the conduction of this study.

## Provenance and peer review

Not commissioned, externally peer-reviewed.

## Declaration of Competing Interest

The authors declare no conflict of interest.
